# Efficacy and safety of mij821 in patients with treatment-resistant depression: Results from a randomized, placebo-controlled, proof-of-concept study

**DOI:** 10.1192/j.eurpsy.2021.897

**Published:** 2021-08-13

**Authors:** N. Ghaemi, A. Sverdlov, R. Shelton, R. Litman

**Affiliations:** 1 Translational Medicine, Neuroscience, Novartis Institutes for Biomedical Research, Cambridge, United States of America; 2 Analytics Gdd / Cd&a Gdd, Novartis Pharmaceuticals Corporation, East Hanover, United States of America; 3 Department Of Psychiatry And Behavioral Neurobiology, The University of Alabama at Birmingham, Birmingham, United States of America; 4 Georgetown University Medical School, CBH Health, LLC, Gaithersburg, United States of America

**Keywords:** MIJ821, depression, efficacy, safety

## Abstract

**Introduction:**

MIJ821 is a novel N-methyl-D-aspartate (NMDA) receptor antagonist, with a potentially low rate of the psychotomimetic side effects that limit the therapeutic utility of ketamine in treatment-refractory depression (TRD).

**Objectives:**

To assess efficacy and safety of MIJ821.

**Methods:**

Adults with TRD (>2 prior treatment failures; Montgomery-Asberg Depression Rating Scale [MADRS], >24) were eligible and were randomized (n=70) to low versus high doses of MIJ821, with two dosing regimens of weekly or biweekly, versus ketamine versus placebo. The primary outcome was change in MADRS total score at 24 hours and final follow up was at 6 weeks.

**Results:**

At 24 hours, adjusted mean differences (ΔAM) versus placebo were –8.25 (p=0.001), –5.71 (p=0.019) and –5.67 (p=0.046) and at 48 h were –7.06 (p=0.013), –7.37 (p=0.013), –11.02 (p=0.019) in the pooled MIJ821 low dose, high dose, and ketamine groups, respectively. At 6 weeks, ΔAM (80% CI) versus placebo on MADRS were –6.46 (–11.8, –1.15); p=0.059 for low dose MIJ821, –5.42 (–10.8, –0.02); p=0.099) for high dose MIJ821, and –5.24 (–10.4, –0.06); p=0.097 for ketamine. Further details on dosing, efficacy, and safety outcomes will be provided.

**Conclusions:**

In this proof-of-concept study, MIJ821 was effective and tolerable in TRD. This study was funded by Novartis. Clinical trial.gov: NCT03756129

**Conflict of interest:**

Employee of Novartis.

